# 30-day healthcare utilisation after discharge from four General Internal Medicine departments in Switzerland: a prospective observational cohort study

**DOI:** 10.1186/s12913-026-14311-w

**Published:** 2026-03-11

**Authors:** Gregor John, Loïc Payrard, Jörg Leuppi, Marco Mancinetti, Daniel Genné, Jacques Donzé

**Affiliations:** 1 Department of Internal Medicine, Neuchâtel Hospital Network, Rue de la Maladière 45, Neuchâtel, CH-2000 Switzerland; 2 Department of Internal Medicine, Geneva University Hospitals (HUG), Rue Gabrielle-Perret-Gentil 4, Geneva, CH-1205 Switzerland; 3 Faculty of Medicine, University of Geneva, Rue Michel-Servet 1, Geneva, Switzerland; 4 Department of Medicine, Neuchâtel Hospital Network, Neuchâtel, Switzerland; 5 Faculty of Medicine, University of Basel, Basel, Switzerland; 6 University Medical Clinic, Baselland Cantonal Hospital, Liestal, Switzerland; 7 Department of Medicine, Fribourg Cantonal Hospital, Fribourg, Switzerland; 8 Medical Education Unit, University of Fribourg, Fribourg, Switzerland; 9 Department of Internal Medicine, Biel Hospital Centre, Biel, Switzerland; 10 Division of Internal Medicine, Inselspital, Bern University Hospital, Bern, Switzerland; 11 Division of Internal Medicine, Lausanne University Hospital (CHUV) and University of Lausanne, Lausanne, Switzerland; 12 Brigham and Women’s Hospital, Harvard Medical School, Boston, MA USA

**Keywords:** Healthcare services, Healthcare utilisation, Hospital discharge, Mortality, Internal Medicine, Hospital readmission, Multimorbidity, HUTIL index, HOSPITAL score

## Abstract

**Aim:**

To assess healthcare utilisation within 30 days of discharge, identify predictors of this utilisation, and examine its association with mortality.

**Methods:**

Adults discharged from four Swiss medicine departments were prospectively followed. The 30-day Healthcare Utilisation (HUTIL) index was calculated with consultations with primary care physicians (PCPs), specialists or at emergency departments (EDs), home visits by nurses and hospital readmissions and compared to the Swiss national values. The one-year age- and sex-adjusted risks of dying for each three-point increase in the HUTIL index were calculated using a Cox regression model.

**Results:**

Of 934 patients included, 78% attended medical consultations, 25% received home-care nurse visits, 12% were readmitted to hospital and 9% consulted at an ED within 30 days of discharge. The median number of healthcare services used per patient (2; IQR 25–75%: 1–13) and HUTIL index scores (3.0; IQR 25–75%: 1.0–8.0) were significantly higher than Switzerland’s 30-day national values of 0.7 (*p* < 0.001) and 1.6 (*p* < 0.001), respectively. High HOSPITAL scores, age, hospital lengths of stay, and number of comorbidities were all associated with healthcare utilisation. Compared to patients with HUTIL index scores of 0–2.9, patients with scores of 3–5.9 (adjusted hazard ratio [aHR] 2.7; 95%CI: 1.1–6.7), 6–8.9 (aHR 5.5; 95%CI: 2.8–10.6), and more than 8.9 (aHR 9.6; 95%CI: 5.2–17.9), all had higher risks of dying within one year.

**Conclusion:**

Hospital discharge is followed by periods of high healthcare services utilisation. HUTIL index scores correlate with patients’ prognoses.

**Supplementary Information:**

The online version contains supplementary material available at 10.1186/s12913-026-14311-w.

## Introduction

Each year, around 20 million patients are discharged from hospitals across Europe and over 34 million in the USA [1, 2]. In the six months following hospitalisation, post-acute care costs around USD 20,000 per patient, mainly comprising home-care services, skilled nursing care, consultations with primary care physicians (PCPs) or specialists, outpatient procedures and, to a lesser extent, emergency department (ED) visits [3]. Because of the high number of hospital discharges, post-hospitalisation care puts a significant burden on healthcare systems worldwide [2]. However, prompt and adequate post-discharge follow-up has the potentially to mitigate the risks of adverse events and avoid costly readmissions [4–6]. To provide the most effective care after an acute care hospitals discharge, we must understand the factors associated with patterns of health service utilisation. Among these, patient-specific comorbidities and factors present during hospital stays are likely to inform us about which healthcare services might be needed after discharge [7].

The Healthcare UTILisation (HUTIL) index has recently been proposed as a single measure of the level of healthcare services utilisation over a defined period [8]. The index sums the different services used over the period, assigning each a weighting based on the ratio of its average cost to the cost of a consultation with a PCP. Thus, these weighting places greater value on the health services required as the severity of an illness increases (from the PCP to the specialist, the ED and finally admission to hospital) [9–11]. It has been shown that European countries with the highest average HUTIL scores also have higher mortality rates [8]. However, as far as we know, this index has never been studied in a clinical setting, and its association with mortality based on individual data has not yet been demonstrated.

Using data collected from the acute general internal medicine departments of four hospitals in Switzerland, we aimed to describe healthcare services utilisation at 30 days post-discharge, reveal factors associated with that use and explore associations between the level of healthcare services utilisation (measured using the HUTIL index) and the risk of death during the 12 months following the index discharge.

## Methods

We used prospective data from the TARGET-READ (Transition cAre intervention targeted to high-risk patiEnts To Reduce rEADmission) study, which recorded patients’ 30-day post-discharge healthcare services utilisation and one-year death and hospital readmission data [10, 12]. The study was conducted in accordance with the principles of Good Clinical Practice and the Declaration of Helsinki.

### Setting and participants

We consecutively included every patient aged 18 or more who was not admitted electively, spent more than 24 h in one of the general internal medicine departments of four mid-sized hospitals in Switzerland (Neuchâtel, Liestal, Bienne and Fribourg) and was discharged alive between July 2017 and March 2018. Patients previously enrolled in the study, who spoke neither French nor German, lived outside Switzerland, did not have a telephone, refused to participate or were unable to understand the study, were excluded. The four participating hospitals’ ethics committees approved the study protocol. Written informed consent was obtained from all participants.

### Outcomes and measurements

The study’s primary outcome was the level of healthcare services utilisation during the first 30 days post-discharge, as measured using the HUTIL index. Secondary outcomes were the 30-day unweighted number of healthcare services used, the proportion of patients using each different healthcare service, the proportion of patients receiving professional home-care support, factors associated with healthcare services utilisation, factors associated with professional home-care support, and death within one year of hospital discharge.

Information on participants’ healthcare utilisation was collected by reviewing their electronic health records and during a scheduled telephone call at 30 days after hospital discharge. This telephone call was dedicated solely to collecting study data and did not interfere with the patient’s care. Healthcare services utilisation was divided into nurses’ visits to patients’ homes, consultations with PCPs or specialists, ED visits, and the number of days spent in hospital after readmission. PCP and specialist consultations were mutually exclusive, with patients reporting the main type of doctor they consulted [9]. The number of days spent in hospital was calculated as the cumulative hospital length of stay (LOS) after one or more readmissions within 30 days of discharge. The exact number of home visits by nurses was not collected. Instead, based on the average home-care nursing invoice charged for the first 30 days after hospital discharge by the canton of Neuchâtel’s main home-care service, we calculated an average of three nurse visits per week. This was consistent with official Swiss data on nursing visits published in 2014 and 2019 [13]. Calculation of the total number of home visits by nurses excluded periods of rehospitalisation and was proportional to the number of days patients spent alive if they died within 30 days of their index discharge. The HUTIL index was calculated as the 30-day weighted sum of healthcare services used (Appendix Methods) [8].

Before discharge from the index hospitalisation, trained study nurses collected information on patient demographics, main diagnoses, comorbidities and LOS, and they calculated patients’ HOSPITAL scores (Appendix Methods) [14]. HOSPITAL scores include data on haemoglobin and sodium levels, discharge from an oncology ward or an active cancer diagnosis, procedures performed during the index hospitalisation, type of admission (urgent or emergent), the number of hospital admissions in the previous year, and lengths of stay ≥ 8 days. Principal diagnoses were classified into nine categories: cardiovascular, thromboembolic, infectious, cerebrovascular, pulmonary, gastroenterological, metabolic, oncological, and miscellaneous diseases. Post-discharge professional home-care support was classified into home-care support for cleaning, buying groceries and eating. Deaths were obtained from death registries and by checking the electronic health records of each participating hospital.

### Statistics

Phase I of the TARGET-READ study aimed to include at least 600 patients with the goal of reaching 70 readmissions (a 12% readmission rate). However, the final sample size was determined by the number of patients discharged during the predefined study period.

Because 30-day HUTIL index scores had a non-normal distribution, the primary analysis used the Wilcoxon signed-rank test to compare the study’s median to the Switzerland’s general population average (1.6; 95%CI: 1.2–1.9) calculated from official national statistics [8]. This analysis was repeated for the secondary outcome of the number of healthcare services used (national average of 0.7; 95%CI: 0.2–1.2) [8]. In a first sensitivity analysis, a modified HUTIL index was built to include measured healthcare utilisation only, without estimated numbers of nurse visits. Patients who reported nurse visits were attributed a single visit. In a second sensitivity analysis, the factors associated with the HUTIL index were stratified by age quartiles and gender.

The factors tested for an association with healthcare services utilisation were age, sex, HOSPITAL score, LOS, number of comorbidities, needs for professional home-care support, living arrangements, type of health insurance (basic or more than basic), and principal diagnosis category at index hospital admission. Those factors were selected based on previous publications [9–11]. Continuous risk factors were categorised into quartiles. The Kruskal–Wallis test was used to test associations between factors and HUTIL index scores, and logistic regression models were used to test associations between each healthcare service (binary dependant variables). Associations were adjusted for age and sex.

The Wilcoxon rank sum test was used to compare the pre-death HUTIL index score of patients who died within 30 days of hospital discharge with that of the 30-day HUTIL index scores of patients alive at the end of this period. Survival analysis beyond 30 days was restricted to patients who had survived that period following hospital discharge. Patients were censored for loss to follow-up, death or after one year of follow-up, whichever occurred first. Thirty-day HUTIL index scores were divided into four categories in increments of three points (0–2.9, 3–5.9, 6–8.9 and ≥ 9). The unadjusted association between HUTIL index score and time to death was analysed using Kaplan–Meier survival analysis and an unweighted, two-tailed log-rank test to compare groups. A Cox regression model was used for age- and sex-adjusted associations. The proportional hazards assumption was verified using Schoenfeld residuals and a visual inspection of the log-minus-log plots.

The significance level was set at 5%, and all analyses were performed using Stata statistical software, version 18.0 (StataCorp LP, College Station, TX, USA).

## Results

**Fig. 1:**
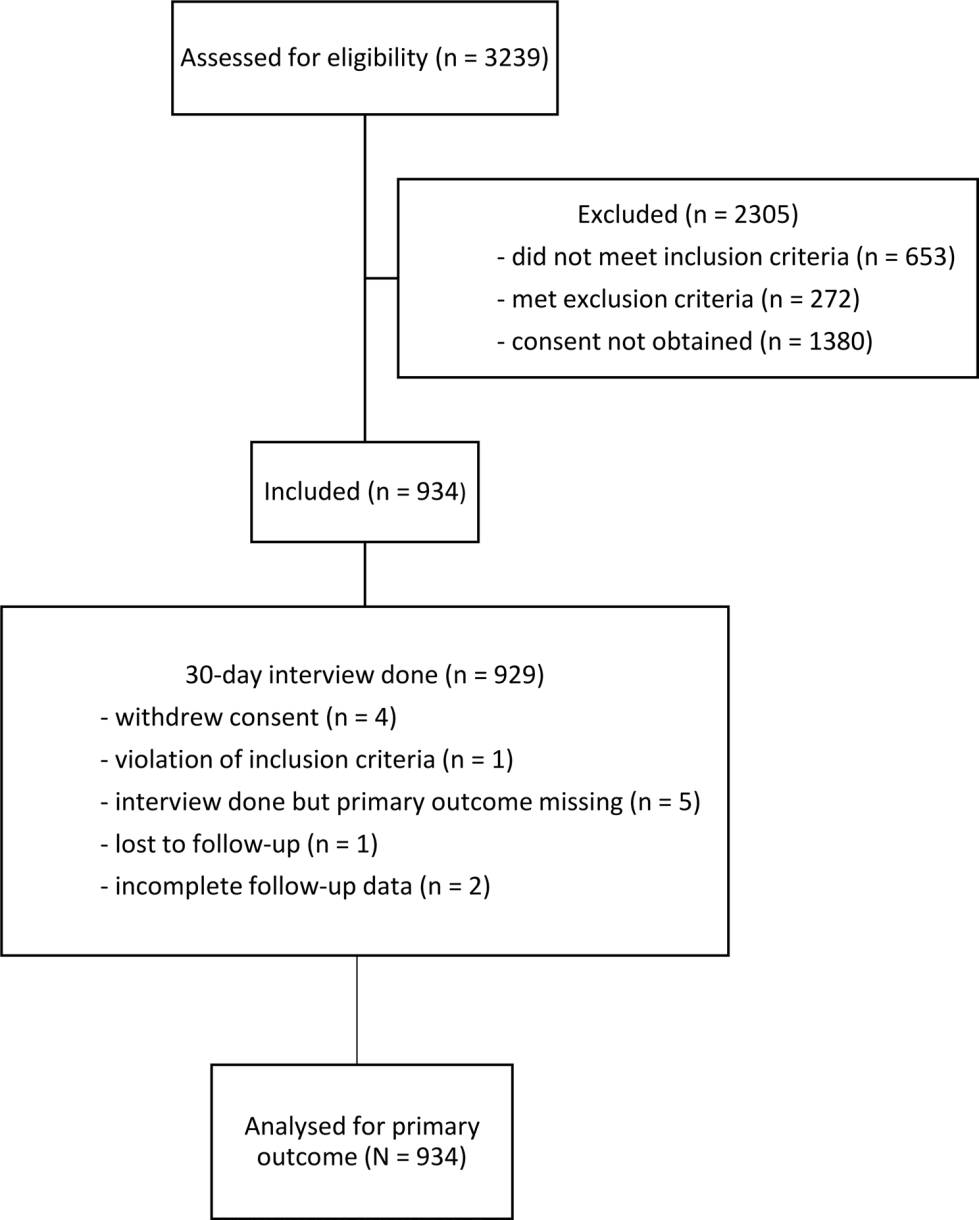
Study flowchart

The analysis included 934 discharged patients (Fig. 1), and Table 1 shows their baseline characteristics.

**Table 1:** Patients’ sociodemographic characteristics

	Total (*N* = 934)
General	
Age (years), median (IQR 25–75%)	71 (58, 80)
Man Woman	526 (56%) 408 (44%)
Living with someone else	614 (67%)
Swiss Other origins	783 (87%) 118 (13%)
*Place of living*	
Home Protected apartment Nursing home Other or unknown	882 (94) 11 (1.2) 35 (3.7) 6 (1%)
*Work situation*	
Active Unemployed Social or invalidity assurance Retired Other or unknown	206 (23%) 16 (2%) 55 (6%) 602 (67%) 22 (2%)
*Health assurance*	
None Basic Basic plus Semi-Private Private	1 (0%) 421 (47%) 256 (28%) 160 (18%) 62 (7%)
*Professional home-care support*	
Support for cleaning Support for buying groceries Support for eating	254 (28%) 92 (10%) 82 (9%)
Comorbid conditions	
Number of comorbid conditions, median (IQR 25–75%)	1 (0, 3)
Chronic heart failure	131 (15%)
Ischaemic heart disease	241 (27%)
Atrial fibrillation	162 (18%)
Peripheral arterial disease	83 (9%)
Diabetes	205 (23%)
Dementia	28 (3%)
Chronic obstructive pulmonary disease	92 (10%)
Active cancer	132 (15%)
Chronic renal disease	189 (21%)
Cirrhosis	29 (3%)
Substance abuse	94 (10%)
Psychiatric disease	92 (10%)
Index hospitalisation	
Main diagnosis: Cardiovascular disease	146 (16%)
Main diagnosis: Thromboembolism	34 (4%)
Main diagnosis: Stroke	55 (6%)
Main diagnosis: Infectious disease	195 (21%)
Main diagnosis: Pulmonary disease	87 (9%)
Main diagnosis: Gastroenterological disease	76 (8%)
Main diagnosis: Metabolic disease	57 (6%)
Main diagnosis: Oncological disease	35 (4%)
LOS of index hospitalisation, median (IQR 25–75%)	6 (4, 9)
Discharge to:	
Home Nursing home	862 (96%) 39 (4%)
Left against medical advice	15 (2%)
HOSPITAL score, median (IQR 25–75%)	3 (2, 5)

IQR = Interquartile range; LOS = length of stay; PCP = primary care physician

### 30-day post-discharge healthcare services utilisation

Only 99 patients (11%) used no healthcare services within 30 days of their discharge. The remaining 835 patients attended a total of 1111 consultations with PCPs, 381 with specialist physicians and 100 at EDs. They had 2834 home visits from nurses and spent 768 days in hospital after readmission. The median 30-day number of healthcare services used per patient was 2 (IQR 25–75%: 1–13), and the median 30-day HUTIL index score was 3.0 (IQR 25–75%: 1.0–8.0), significantly higher than Switzerland’s 30-day national averages of 0.7 (*p* < 0.001) and 1.6 (*p* < 0.001), respectively. These differences persisted in sensitivity analyses.

### Factors associated with healthcare services

Thirty-day HUTIL index scores increased along with greater HOSPITAL scores, LOS, age and the number of comorbidities (Table 2). Scores were also higher among patients who lived alone, needed professional home-care support or had an oncological disease as their index hospitalisation’s principal diagnosis. These findings were similar in sensitivity analyses (eTable 1, and 2).

**Table 2:** 30-day health care utilisation after index hospitalisation measured using the HUTIL index and its association with potential risk factors

	HUTIL Index	
	Median (IQR 25–75%)	*P*-value*
Men Women	2.0 (1.0–7.5) 3.0 (1.0–8.5)	0.23
Age < 60 years Age 61–70 years Age 71–80 years Age > 80 years	2.0 (1.0–5.0) 3.0 (1.0–7.5) 3.0 (1.0–8.5) 6.5 (1.0–8.5)	< 0.01
Living alone Living with someone else	4.0 (1.0–8.5) 2.0 (1.0–7.5)	< 0.01
Formal support nb = 0 Formal support nb = 1 Formal support nb = 2 Formal support nb = 3	2.0 (1.0–6.0) 7.5 (2.0–9.5) 7.5 (4.3–10.5) 7.5 (6.5–9.5)	< 0.01
HOSPITAL score 0–1 HOSPITAL score 2–3 HOSPITAL score 4–5 HOSPITAL score > 5	2.0 (1.0–4.0) 3.0 (1.0–7.5) 4.0 (2.0–8.5) 7.5 (2.0–10.5)	< 0.01
LOS 1–3 days LOS 4–5 days LOS 6–8 days LOS > 8 days	2.0 (1.0–4.0) 2.0 (1.0–6.5) 3.0 (1.0–8.2) 6.5 (2.0–9.0)	< 0.01
Comorbidities nb = 0 Comorbidities nb = 1 Comorbidities nb = 2 Comorbidities nb > 2	2.0 (1.0–4.0) 3.0 (1.0–7.5) 4.0 (2.0–8.5) 6.5 (2.0–9.0)	< 0.01
Basic health insurance Private/semi-private	3.0 (1.0–8.5) 3.0 (1.0–7.5)	0.43
Oncological disease^†^	8.0 (2.0–26)	< 0.01
Cardiovascular disease^†^	3.0 (1.0–7.5)	0.81
Thromboembolism^†^	2.0 (1.0–7.5)	0.33
Stroke^†^	2.0 (1.0–4.0)	0.05
Pulmonary disease^†^	3.0 (1.0–8.5)	0.45
Infectious disease^†^	3.0 (1.0–8.0)	0.40
Abdominal disease^†^	4.0 (1.0–8.5)	0.27
Metabolic disease^†^	2.0 (2.0–6.5)	0.63

ED = Emergency department; HUTIL = Healthcare Utilisation; LOS = Length of stay

* Kruskal–Wallis test; ^†^ Main diagnosis of the index hospital admission

**Fig. 2:**
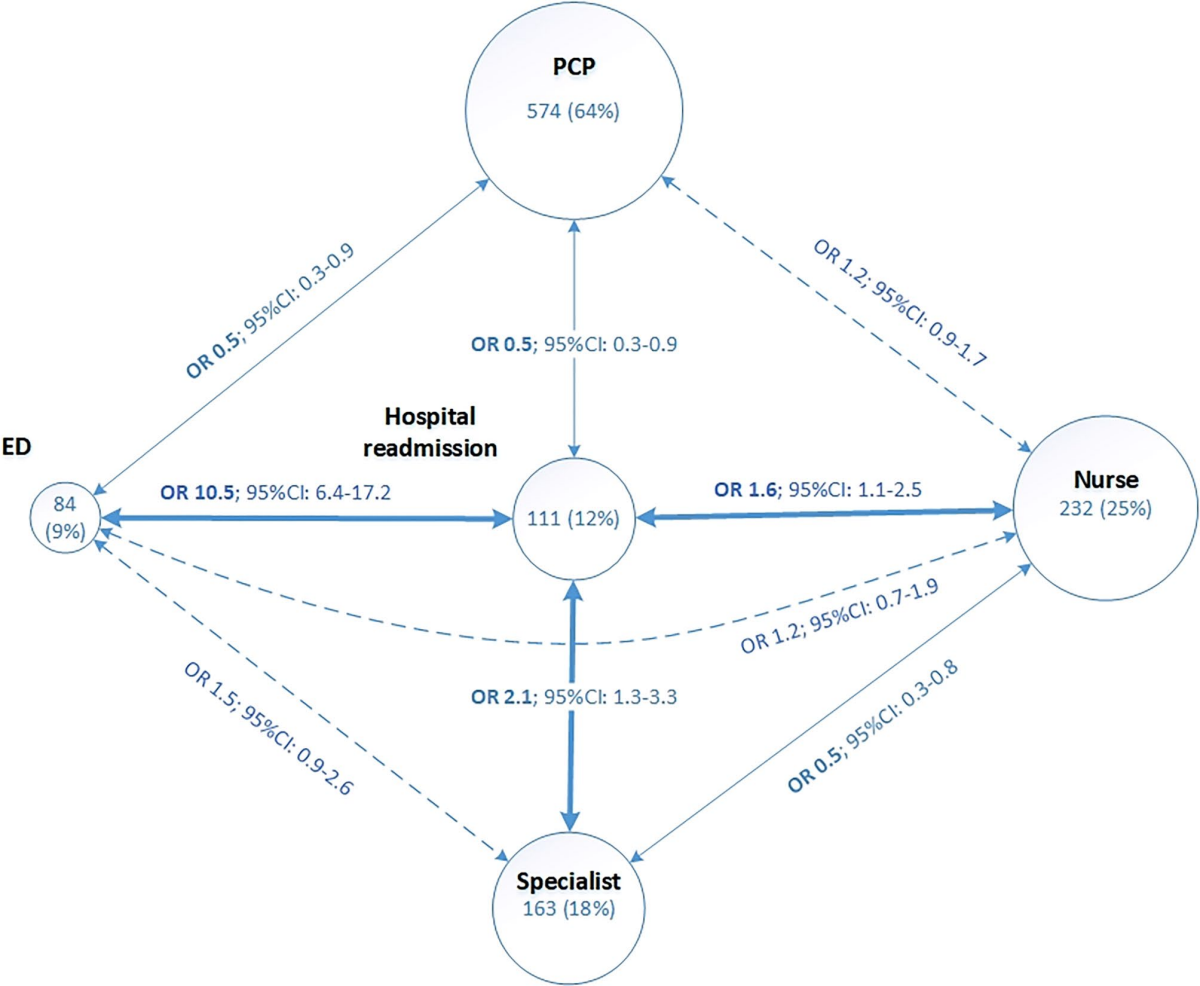
Interactions between healthcare services used within 30 days of the patient’s index hospitalisation. Circle sizes represent the number of patients (percentage) who had at least one consultation, visit or admission. The thicker arrows represent positive associations, the thinner arrows represent negative associations, and the dashed arrows represent non-statistically significant associations. Since medical consultations were dichotomised between those with PCPs or specialists (mutually exclusive), no comparison between these two types of consultations was performed. ED = emergency department; PCP = Primary care physician; OR = odds ratio

Different types of health services utilisation were interdependent (Fig. 2). For example hospital readmission was associated with home visits by a nurse and consultations with a specialist or at an ED, but it was inversely associated with consultations with a PCP. Findings relative to each healthcare service used are presented in eTable 3. Higher HOSPITAL scores and longer LOS were associated with home visits by nurses, consultations with specialists or at an ED and hospital readmission risk. Patients’ older age and more formal support were associated with home visits by nurses and fewer post-discharges consultation with specialists. Results concerning factors associated with professional home support are presented in eTables 4–6.

### Post-discharge healthcare services utilisation and mortality

Twenty-two (2.4%) patients died within 30 days of hospital discharge, after a median of 17 days (IQR 25–75%: 11–23). Their median pre-death HUTIL index score was significantly higher than the median 30-day HUTIL index score of patients still alive at 30 days: 15.0 (IQR 25–75%: 6.0–24.0) and 3.0 (IQR 25–75%: 1.0–8.0, *p* < 0.001), respectively.

**Fig. 3:**
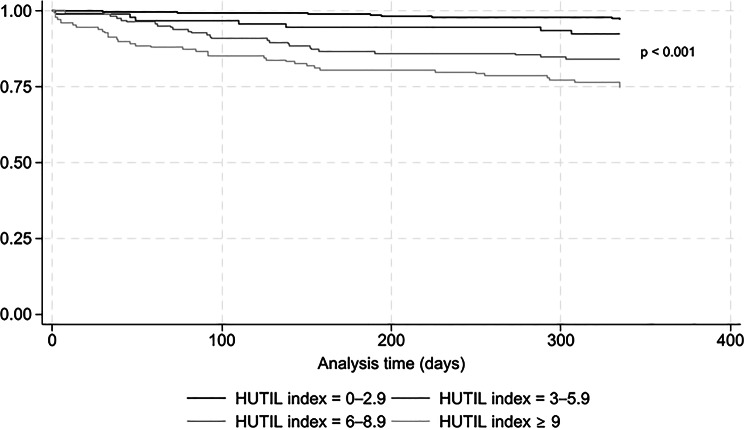
Kaplan–Meier survival estimates using 30-day HUTIL index categories. The zero point on the horizontal axis was set at 30 days after discharge, and only patients alive at that time were included in the analysis

Among the 912 (98.3%) patients who survived the first 30 days after discharge, 91 (10.0%) died in the following 11 months. The 30-day HUTIL index score was associated with the risk of death (Fig. 3). Using a Cox proportional hazards model, patients with HUTIL index scores from 3 to 5.9 (HR 2.6; 95%CI: 1.1–6.5), from 6 to 8.9 (HR 5.8; 95%CI: 3.0–11.2), and > 8.9 (HR 10.0; 95%CI: 5.4–18.5), had a greater risk of dying than patients with HUTIL index scores from 0 to 2.9. These associations persisted in age- and sex-adjusted Cox regression models with hazard ratios (HRs) of 2.7 (95%CI: 1.1–6.7), 5.5 (95%CI: 2.8–10.6), and 9.6 (95%CI: 5.2–17.9), respectively. These associations were confirmed in sensitivity analyses (eTable 4).

## Discussion

The present cohort study found a high level of healthcare services utilisation within 30 days of discharge from four acute internal medicine departments in Switzerland. The level of healthcare services used was three times the national average of healthcare utilisation [8]. Patterns and numbers of services used after discharge were associated with information gathered at discharge, such as the patient’s LOS, number of comorbidities and HOSPITAL score. A greater level of healthcare services utilisation (as measured using the HUTIL index) was associated with mortality.

Patterns of healthcare use after hospitalisation in a general internal medicine ward are less studied than the transitional care required for specific diseases (e.g. diabetes, heart failure, chronic obstructive pulmonary disease) [4–6]. Our study provided insight into the factors associated with this population’s post-discharge healthcare services utilisation. Notably, the higher the HOSPITAL score, the greater the risk of an unplanned readmission, a consultation at an ED or with a specialist, a visit from a nurse and more need for professional home-care support. The HOSPITAL score could thus be valuable and easily calculated during a patient’s index hospitalisation to predict their post-discharge healthcare needs or utilisation. It is noteworthy that, except for oncological diseases, the primary diagnosis for the index hospitalisation was a poor predictor of who would subsequently use more healthcare services or professional home-care support. Similarly, although sex’s influence on care is well recognised [15], we found no sex differences in the number of consultations with a PCP, a specialist or at an ED or in the number of unplanned readmissions observed in this cohort.

High post-discharge healthcare services utilisation was associated with mortality. Moreover, even though patients who died within 30 days of discharge had lived only a median of 17 further days, they had higher HUTIL scores than those who survived to 30 days—roughly twice as long. This is consistent with the observation that the period preceding death consumes a large amount of health resources, with more than 94% of patients requiring medical consultations and 61% being hospitalised for a mean LOS of 28 days [16, 17]. Furthermore, according to the Swiss Health Observatory, only 19% of the population die at home, 37% in hospital and 44% in care facilities (often after a hospitalisation) [18].

The association between HUTIL index scores and mortality is obviously not causal. Rather, scores are indicators of the severity of patients’ underlying diseases or frailty. By placing greater importance on the healthcare services utilised as the severity of an illness increases (PCPs, specialists, EDs and finally hospitalisation), the HUTIL index highlights the complexity of the care patients require. Indeed, in the study that established this score, the association between healthcare utilisation and mortality was only revealed once the scores were calculated, but could not be discerned by simply summing the number of health services used [8]. Finally, it is interesting to note that HOSPITAL scores, age and the number of comorbidities are associated with HUTIL index scores, and have all previously been found to be associated with mortality [10, 19].

The HUTIL index, therefore, could be of interest to researchers and other stakeholders in the field of health services utilisation (e.g. policymakers, health sector leaders) as it correlates with the number of healthcare services used, their economic burden and important health outcomes (e.g. mortality). For example, the index could be used to compare healthcare utilisation after hospital discharge in different contexts and countries, or over long periods of time. This comparison would be more generalisable than using healthcare expenditure, which varies considerably around the world and over time [8]. The HUTIL index could also help analyse a particular intervention’s effects on resource utilisation and the complexity of care. For example, an intervention targeting patients at a high risk of post-discharge health services utilisation (e.g. high HOSPITAL scores) could offer them a more intense post-discharge follow-up in the form of consultations with PCPs and frequent visits by nurses (to monitor weight, treatment compliance or side effects) with the aim of reducing the risk of hospital readmission [20]. Post-intervention HUTIL index scores should be lower, as the overall complexity of care should have been reduced (fewer readmissions or ED visits), despite the absolute number of services utilised being the same or even greater (i.e. days of hospitalisation saved are offset by more nurse visits and PCP consultations). In this situation, the HUTIL index would be better correlated with benefits for patients and the economy than would be the number of services utilised. Nevertheless, this example warrants two caveats. Firstly, although the patients in our cohort who consulted a PCP were less likely to be readmitted to hospital or to visit an ED, precisely what constitutes an optimal transition of care has yet to be defined, especially one that would prevent these adverse outcomes—it will likely depend on multiple interventions [21–23], admission variables [11], and patient-specific comorbidities and diagnoses [7]. Secondly, although HOSPITAL scores, the number of comorbidities and LOS could be used to identify patients with greater needs, further research is needed to determine whether using these indicators to tailor post-discharge care actually has an impact.

The present study had some limitations. First, as an observational study, no inferences can be made as to the causative nature of the associations revealed. Second, as the study nurses contacted the patients after discharge to check whether any follow-up consultations had been attended, healthcare services utilisation may have been influenced by the study’s very nature. However, we suggest that this risk was limited since the study nurses did not interact with the medical teams involved in the hospital discharges. Third, the study was conducted among a specific population of patients following their admission to acute internal medicine departments in Swiss hospitals. In Switzerland, healthcare services are unrestricted and all patients are insured. Our observations might not be generalisable to other countries where access to healthcare access might be hampered by particular issues or by the need for significant personal expenditure. Furthermore, the high rate of care utilisation observed in this study may have been boosted by the inclusion of significant numbers of older adults, who usually require more extensive care. Adding to this, many hospitals in Switzerland have introduced transitional care programmes that have increased the number of case managers and pre-discharge communication between hospitals and PCPs, community nurses and relatives, thus better anticipating post-discharge needs. Finally, the exact number of nurses’ visits to patients’ homes was not measured but rather extrapolated from the country’s official mean. This may have diminished the accuracy of the calculation of the number of healthcare services utilised and the HUTIL index scores. Nevertheless, calculations were applied homogeneously to all patients, and a nurse’s visit has a low weighting in the HUTIL index [8]. Furthermore, our sensitivity analysis attributing a single visit to patients known to have had home nurse visits did not alter the results.

## Supplementary Information

Below is the link to the electronic supplementary material.

Supplementary Material 1

## Data Availability

The datasets generated and/or analysed during the current study are not publicly available but can be obtained from the corresponding author on reasonable request.

## References

[1] WHO. WHO European health information at your fingertips. 2021; Available from: https://gateway.euro.who.int/en/indicators/hfa_535-6011-number-of-all-hospital-discharges/.

[2] Hall MJ, DeFrances CJ, Williams SN, Golosinskiy A, Schwartzman A. National Hospital Discharge Survey: 2007 summary. Natl Health Stat Rep. 2010;29:1–20. 4. 21086860

[3] Gardner R, Li Q, Baier RR, Butterfield K, Coleman EA, Gravenstein S. Is implementation of the care transitions intervention associated with cost avoidance after hospital discharge? J Gen Intern Med. 2014;29(6):878–84. 24590737 10.1007/s11606-014-2814-0 PMC4026506

[4] Garnica P. Transition of Care for Patients with Diabetes. Curr Diabetes Rev. 2017;13(3):263–79. 27881054 10.2174/1573399813666161123104407

[5] Naylor MD, Brooten DA, Campbell RL, Maislin G, McCauley KM, Schwartz JS. Transitional care of older adults hospitalized with heart failure: a randomized, controlled trial. J Am Geriatr Soc. 2004;52(5):675–84. 15086645 10.1111/j.1532-5415.2004.52202.x

[6] Ridwan ES, Hadi H, Wu YL, Tsai PS. Effects of Transitional Care on Hospital Readmission and Mortality Rate in Subjects With COPD: A Systematic Review and Meta-Analysis. Respir Care. 2019;64(9):1146–56. 31467155 10.4187/respcare.06959

[7] Donzé J, Lipsitz S, Bates DW, Schnipper JL. Causes and patterns of readmissions in patients with common comorbidities: retrospective cohort study. BMJ. 2013;347:f7171. 24342737 10.1136/bmj.f7171 PMC3898702

[8] John G, Rebell D, Donze J. Development of a healthcare utilization index to compare patients worldwide: A cross-sectional study. BMC Health Serv Res. 2025;25(1):895. 40604968 10.1186/s12913-025-13029-5 PMC12224835

[9] John G, Payrard L, Donzé J. Associations between post-discharge medical consultations and 30-day unplanned hospital readmission: A prospective observational cohort study. Eur J Intern Med. 2022;99:57–62. 35034807 10.1016/j.ejim.2022.01.013

[10] Liechti FD, Butikofer L, Mancinetti M, Leuppi JD, Genne D, John G, et al. Factors associated with one-year mortality after hospital discharge: A multicenter prospective cohort study. PLoS ONE. 2023;18(8):e0288842. 37556442 10.1371/journal.pone.0288842 PMC10411790

[11] Donze J, John G, Genne D, Mancinetti M, Gouveia A, Mean M, et al. Effects of a Multimodal Transitional Care Intervention in Patients at High Risk of Readmission: The TARGET-READ Randomized Clinical Trial. JAMA Intern Med. 2023;183(7):658–68. 37126338 10.1001/jamainternmed.2023.0791 PMC10152373

[12] Gouveia A, Mancinetti M, Genne D, Mean M, John G, Butikofer L, et al. Effectiveness of transition care intervention targeted to high-risk patients to reduce readmissions: study protocol for the TARGET-READ multicenter randomized-controlled trial. Healthcare (Basel). 2023;11(6). 10.3390/healthcare11060886 PMC10048511 36981543

[13] Office Fédéreal de la Statistique. Spitex: Aide à domicile par canton. 2023; Available from: https://www.bfs.admin.ch/bfs/fr/home/statistiques/sante/systeme-sante/aide-soins-domicile.assetdetail.23566172.html.

[14] Donze JD, Williams MV, Robinson EJ, Zimlichman E, Aujesky D, Vasilevskis EE, et al. International Validity of the HOSPITAL Score to Predict 30-Day Potentially Avoidable Hospital Readmissions. JAMA Intern Med. 2016;176(4):496–502. 26954698 10.1001/jamainternmed.2015.8462 PMC5070968

[15] Mauvais-Jarvis F, Bairey Merz N, Barnes PJ, Brinton RD, Carrero JJ, DeMeo DL, et al. Sex and gender: modifiers of health, disease, and medicine. Lancet. 2020;396(10250):565–82. 32828189 10.1016/S0140-6736(20)31561-0 PMC7440877

[16] Bahler C, Signorell A, Reich O. Health Care Utilisation and Transitions between Health Care Settings in the Last 6 Months of Life in Switzerland. PLoS ONE. 2016;11(9):e0160932. 27598939 10.1371/journal.pone.0160932 PMC5012658

[17] Reich O, Signorell A, Busato A. Place of death and health care utilization for people in the last 6 months of life in Switzerland: a retrospective analysis using administrative data. BMC Health Serv Res. 2013;13:116. 23530717 10.1186/1472-6963-13-116 PMC3623664

[18] Füglister-Dousse S, Pellegrini S, (OBSAN). {Les trajectoires de fin de vie des personnes âgées}. Observatoire suisse de la santé. 2018. Available from: https://dam-api.bfs.admin.ch/hub/api/dam/assets/7427474/master.

[19] Sundararajan V, Henderson T, Perry C, Muggivan A, Quan H, Ghali WA. New ICD-10 version of the Charlson comorbidity index predicted in-hospital mortality. J Clin Epidemiol. 2004;57(12):1288–94. 15617955 10.1016/j.jclinepi.2004.03.012

[20] Naylor MD, Brooten D, Campbell R, Jacobsen BS, Mezey MD, Pauly MV, et al. Comprehensive discharge planning and home follow-up of hospitalized elders: a randomized clinical trial. JAMA. 1999;281(7):613–20. 10029122 10.1001/jama.281.7.613

[21] Rennke S, Nguyen OK, Shoeb MH, Magan Y, Wachter RM, Ranji SR. Hospital-initiated transitional care interventions as a patient safety strategy: a systematic review. Ann Intern Med. 2013;158(5 Pt 2):433–40. 23460101 10.7326/0003-4819-158-5-201303051-00011

[22] Hansen LO, Young RS, Hinami K, Leung A, Williams MV. Interventions to reduce 30-day rehospitalization: a systematic review. Ann Intern Med. 2011;155(8):520–8. 22007045 10.7326/0003-4819-155-8-201110180-00008

[23] Leppin AL, Gionfriddo MR, Kessler M, Brito JP, Mair FS, Gallacher K, et al. Preventing 30-day hospital readmissions: a systematic review and meta-analysis of randomized trials. JAMA Intern Med. 2014;174(7):1095–107. 24820131 10.1001/jamainternmed.2014.1608 PMC4249925

