# Supplementary material for: 30-day healthcare utilisation after discharge from four General Internal Medicine departments in Switzerland: a prospective observational cohort study
Source: BMC Health Serv Res. 2026 Mar 11;26:547. doi: 10.1186/s12913-026-14311-w (PMC13093932; doi:10.1186/s12913-026-14311-w)
Supplement: Supplementary file 1 — Supplementary Material 1 [file 12913_2026_14311_MOESM1_ESM.docx]

30-day healthcare utilisation after discharge from four General Internal Medicine departments in Switzerland: a prospective observational cohort study

**G. John**, **L. Payrard**, **J. Leuppi**, **M. Mancinetti**, **D. Genné**, **J. Donzé**,

Appendix

| **eMethod:** HOSPITAL score and 30 days post discharge HUTIL Index calculation | p 2 |
| --- | --- |
| **eTable1:** 30-day healthcare utilization and their association with risk factors (Modified HUTIL index, sensitivity analysis 1) | p 3 |
| **eTable2:** 30-day healthcare utilization and their association with risk factors (stratified by age quartiles and sex, sensitivity analysis 2) | p 4 |
| **eTable3:** 30-day healthcare utilisation after index hospitalisation and their association with potential risk factors. | p 5-6 |
| **eTable4:** 30-day formal support utilization and their association with risk factors | p 7 |
| **eTable5:** 30-day adjusted Odd ratios for formal support utilization | p 8 |
| **eTable6:** 30-day formal support utilization and its association with main diagnosis category of the index hospitalization. | p 9 |
| **eTable7:** Modified HUTIL index* and mortality beyond 30-day after discharge (sensitivity analysis) | p 10 |

**eMethod:** HOSPITAL score and 30 days post discharge HUTIL Index calculation

HOSPITAL score

The HOSPITAL score includes haemoglobin and sodium levels, discharge from an oncology ward or a diagnosis of active cancer, procedure performed during the index hospitalization, type of admission (urgent or emergent), number of hospital admission(s) in the previous year, and length of stay ≥ 8 days [10].

HUTIL Index

The HUTIL index was calculated as the 30-day weighted sum of health services used [5]: PCP + 2 x specialist consultations + 4 x ED visits + 8 x the number of days spent in hospital + 0.5 x nurse visits. European standards for annual healthcare utilization and HUTIL index have been published [5].

| **eTable 1:** 30-day health care utilization after index hospitalization and their association with potential risk factors (sensitivity analyses). | | |
| --- | --- | --- |
|  | **Modified HUTIL Index*** | |
|  | Median (IQR25-75) | P  Value |
| Factors associated with the modified HUTIL index* | | |
| Men  Women | 2.0 (1.0-4.0)  2.0 (1.0-4.0) | 0.96 |
| Age <60 years  Age 61-70 years  Age 71-80 years  Age >80 years | 2.0 (1.0-4.0)  2.0 (1.0-4.5)  2.0 (1.0-3.5)  2.0 (1.0-4.0) | 0.11 |
| Living alone  Living with someone | 2.0 (1.0-4.0)  2.0 (1.0-4.0) | 0.31 |
| Formal support nb=0  Formal support nb=1  Formal support nb=2  Formal support nb=3 | 2.0 (1.0-4.0)  2.5 (1.5-4.5)  2.5 (1.5-4.8)  1.5 (0.5-4.5) | 0.02 |
| HOSPITAL score 0-1  HOSPITAL score 2-3  HOSPITAL score 4-5  HOSPITAL score >5 | 2.0 (1.0-3.0)  2.0 (1.0-4.0)  2.0 (1.5-4.0)  4.0 (1.5-8.0) | <0.01 |
| LOS 0-3 days  LOS 4-5 days  LOS 6-8 days  LOS >8 days | 2.0 (1.0-3.0)  2.0 (1.0-4.0)  2.0 (1.0-4.5)  2.5 (1.5-4.5) | <0.01 |
| Comorbidities nb=0  Comorbidities nb=1  Comorbidities nb=2  Comorbidities nb>2 | 2.0 (1.0-3.0)  2.0 (1.0-4.0)  2.0 (1.0-4.3)  2.0 (1.0-4.5) | <0.01 |
| Standard Insurance  Private / semi private | 2.0 (1.0-4.0)  2.0 (1.0-4.0) | 0.36 |
| Oncologic disease | 6.0 (1.0-22.5) | <0.01 |
| Cardiovascular | 2.0 (1.0-4.0) | 0.18 |
| Thrombo- embolism | 2.0 (1.0-4.0) | 0.84 |
| Stroke | 2.0 (1.0-2.5) | 0.29 |
| Pulmonary disease | 2.0 (1.0-4.5) | 0.64 |
| Infectious disease | 2.0 (1.0-4.0) | 0.29 |
| Abdominal disease | 2.0 (1.0-4.5) | 0.67 |
| Metabolic disease | 2.0 (1.0-4.0) | 0.46 |
| Modified HUTIL index* and mortality beyond 30-day after discharge | | |
| Alive at 30 days  Dead at 30 days | 2.0 (1.0-4.0)  12.5 (4.5-21.5) | <0.01 |
| * HUTIL index including only one visit for patients with known nurse visits at their home.  HUTIL: Healthcare Utilisation; LOS: Length of hospital stay | | |

| **eTable 2**: 30-day health care utilization (HUTIL) index and their association with potential risk factors stratified by age and sex. | | | | | | |
| --- | --- | --- | --- | --- | --- | --- |
|  | **By age strata** | | | | **By sex** | |
|  | **Age <60** | **Age 61-70** | **Age 71-80** | **Age >80** | **Men** | **Women** |
| HOSPITAL score 0-1  HOSPITAL score 2-3  HOSPITAL score 4-5  HOSPITAL score >5  *P-*value* | 2 (1-4)  2 (1-4)  2 (1-8)  8 (4-18)  P <0.001 | 2 (1-4)  3 (2-6.5)  4 (2-8.5)  7.5 (1-11)  P=0.020 | 2 (1-4)  2 (1-6.5)  4 (2-8.5)  8.5 (2-10.5)  P <0.001 | 2 (1-8)  7 (2-8)  6.5 (2-9.5)  7.5 (4-9.5)  P=0.023 | 2 (1-4)  2 (1-7.5)  4 (2-8.5)  8 (2-16)  P <0.001 | 2 (1-6)  4 (2-8.5)  4 (2-8.5)  7.5 (3-10.5)  P <0.001 |
| LOS 0-3 days  LOS 4-5 days  LOS 6-8 days  LOS >8 days  *P-*value* | 2 (1-4)  2 (1-4)  2 (1-6)  2 (1-8.5)  P=0.058 | 2 (1-6)  2 (1-6.5)  2 (1-7.5)  5 (2-10.5)  P=0.18 | 2 (1-6.5)  2 (1-4)  2.5 (1-8.5)  6.5 (2-8.5)  P=0.011 | 1.5 (1-5.2)  4 (1-8.5)  6.5 (2-9)  7.5 (4-10.5)  P <0.001 | 2 (1-4)  2 (1-4)  3 (1-8)  6.5 (2-8.5)  P <0.001 | 2 (1-4)  2 (1-7.5)  4 (1-8.5)  7.5 (2-10)  P <0.001 |
| Comorbidities nb=0  Comorbidities nb=1  Comorbidities nb=2  Comorbidities nb=3  *P-*value* | 2 (1-3)  2 (1-5)  3.5 (1-10)  5 (2-10.5)  P <0.001 | 2 (1-4)  4 (1-8)  3 (1-7.5)  5.5 (2-8.5)  P=0.034 | 2 (1-3)  3 (1-8)  6 (2-8.5)  6.5 (2-8.5)  P <0.001 | 2 (0.5-8.5)  2 (1-7.5)  6.5 (2-8.5)  7.5 (2-10)  P=0.018 | 2 (1-4)  2 (1-7.5)  4 (2-8.5)  6.5 (2-8.5)  P <0.001 | 2 (1-5)  3 (1-8)  6.5 (2-8.5)  7.5 (2-10.5)  P <0.001 |
| Living with someone Living alone  *P-*value* | 2 (1-4)  2 (1-7.5)  P=0.89 | 3 (1-7.5)  4 (2-8.5)  P=0.23 | 2 (1-8.5)  4 (2-8.5)  P=0.095 | 4 (1-8.5)  7.5 (2-8.5)  P=0.24 | 2 (1-7.5)  3 (1-8.5)  P=0.18 | 2 (1-7.5)  6.5 (1-8.5)  P=0.029 |
| Formal support nb=0  Formal support nb=1  Formal support nb=2  Formal support nb=3  *P-*value* | 2 (1-4)  5.5 (2-10)  8.5 (8-9.5)  8.5 (4-25)  P=0.003 | 2 (1-5.5)  7.5 (3-10)  6.5 (5-7.5)  7.5 (6.5-10.5)  P=0.002 | 2 (1-7)  6.5 (2-8.5)  9.5 (6.5-25)  6.5 (6.5-7.5)  P <0.001 | 2 (1-7.5)  7.5 (2-9.5)  7.5 (3-8.5)  8 (7-10)  P <0.001 | 2 (1-6)  6.5 (2-8.5)  7.5 (5-10.5)  8.5 (6.5-10.5)  P <0.001 | 2 (1-6)  7.5 (2-10)  8.5 (4-11)  7.5 (6.5-8.5)  P <0.001 |
| No oncologic disease  Oncologic disease  *P-*value* | 2 (1-4)  8 (1-25)  P=0.11 | 2 (1-7.5)  9.7 (1-25)  P=0.050 | 2.5 (1-8.5)  18.5 (7.7-53)  P=0.018 | 6.5 (1-8.5)  8.5 (6-58)  P=0.29 | 2 (1-7.5)  10.5 (1-26)  P=0.008 | 3 (1-8.5)  7.5 (2-26)  P=0.06 |
| Nb: number; LOS = length of hospital stays. * Kruskal–Wallis test. | | | | | | |

| **Table 3:** 30-day health care utilisation after index hospitalisation and their association with potential risk factors. | | | | | | | |
| --- | --- | --- | --- | --- | --- | --- | --- |
|  | **HUTIL Index** | | **Consultation with a PCP** | **Consultation with a specialist** | **Home visit by a nurse** | **Consultation at an ED** | **Hospitalisation** |
|  | Median (IQR 25–75%) | *P-*value* | OR (95%CI) | OR (95%CI) | OR (95%CI) | OR (95%CI) | OR (95%CI) |
| Men  Women | 2.0 (1.0–7.5)  3.0 (1.0–8.5) | 0.23 | 1  1.2 (0.9–1.5) | 1  0.8 (0.6–1.2) | 1  1.4 (1.1–1.9) ^†^ | 1  1.0 (0.6–1.5) | 1  1.0 (0.7–1.5) |
| Age < 60 years  Age 61–70 years  Age 71–80 years  Age > 80 years | 2.0 (1.0–5.0)  3.0 (1.0–7.5)  3.0 (1.0–8.5)  6.5 (1.0–8.5) | < 0.01 | 1  1.1 (0.7–2.6)  1.2 (0.8–1.7)  1.2 (0.8–1.8) | 1  1.1 (0.7–1.7)  0.6 (0.4–0.9) ^‡^  0.3 (0.2–0.5) ^‡^ | 1  2.7 (1.5–4.9) ^‡^  5.7 (3.3–9.8) ^‡^  10.1 (6.4–18.8) ^‡^ | 1  1.0 (0.6–1.8)  0.7 (0.4–1.3)  0.7 (0.4–1.3) | 1  1.4 (0.8–2.6)  1.3 (0.8–2.4)  1.8 (1.0–3.1) ^‡^ |
| Living alone  Living with someone else | 4.0 (1.0–8.5)  2.0 (1.0–7.5) | < 0.01 | 1  1.1 (0.8–1.5) | 1  1.1 (0.8–1.6) | 1  0.3 (0.2–0.5) ^‡^ | 1  0.9 (0.6–1.5) | 1  1.0 (0.6–1.5) |
| Formal support nb = 0  Formal support nb = 1  Formal support nb = 2  Formal support nb = 3 | 2.0 (1.0–6.0)  7.5 (2.0–9.5)  7.5 (4.3–10.5)  7.5 (6.5–9.5) | < 0.01 | 1  1.0 (0.7–1.5)  1.1 (0.6–2.1)  0.8 (0.4–1.4) | 1  0.9 (0.6–1.5)  0.7 (0.3–1.7)  0.4 (0.2–1.1) | 1  5.8 (3.9–8.6) ^‡^  13.9 (7.6–25.9) ^‡^  27.6 (14.0–54.3) ^‡^ | 1  1.3 (0.8–2.4)  1.8 (0.8–4.1)  0.8 (0.3–2.3) | 1  1.6 (0.9–2.7)  2.5 (1.2–5.0) ^‡^  1.4 (0.6–3.1) |
| HOSPITAL score 0–1  HOSPITAL score 2–3  HOSPITAL score 4–5  HOSPITAL score > 5 | 2.0 (1.0–4.0)  3.0 (1.0–7.5)  4.0 (2.0–8.5)  7.5 (2.0–10.5) | < 0.01 | 1  0.8 (0.5–1.1)  0.9 (0.6–1.2)  0.4 (0.3–0.6) ^‡^ | 1  2.0 (1.2–3.3) ^‡^  1.4 (0.8–2.1)  3.0 (1.9–4.8) ^‡^ | 1  2.1 (1.3–3.4) ^‡^  2.7 (1.8–4.0) ^‡^  4.2 (2.7–6.5) ^‡^ | 1  1.4 (0.6–2.9)  1.9 (1.1–3.5) ^‡^  3.2 (1.8–6.0) ^‡^ | 1  1.6 (0.8–3.2)  2.2 (1.3–3.7) ^‡^  3.2 (1.8–5.6) ^‡^ |
| LOS 1–3 days  LOS 4–5 days  LOS 6–8 days  LOS > 8 days | 2.0 (1.0–4.0)  2.0 (1.0–6.5)  3.0 (1.0–8.2)  6.5 (2.0–9.0) | < 0.01 | 1  0.8 (0.5–1.1)  1.2 (0.8–1.7)  1.0 (0.7–1.5) | 1  1.7 (1.0–2.8) ^‡^  1.3 (0.8–2.1)  1.0 (0.6–1.7) | 1  1.1 (0.6–2.0)  2.3 (1.4–3.9) ^‡^  5.9 (3.6–9.7) ^‡^ | 1  2.9 (1.2–7.1) ^‡^  2.7 (1.1–6.4) ^‡^  3.4 (1.5–8.0) ^‡^ | 1  1.4 (0.7–2.8)  2.2 (1.2–4.2) ^‡^  2.0 (1.0–3.8) ^†^ |
| Comorbidities nb = 0  Comorbidities nb = 1  Comorbidities nb = 2  Comorbidities nb > 2 | 2.0 (1.0–4.0)  3.0 (1.0–7.5)  4.0 (2.0–8.5)  6.5 (2.0–9.0) | < 0.01 | 1  0.8 (0.5–1.1)  0.8 (0.5–1.2)  1.1 (0.8–1.6) | 1  1.8 (1.1–2.8) ^‡^  1.8 (1.1–2.9) ^‡^  0.8 (0.5–1.4) | 1  3.6 (2.0–6.6) ^‡^  6.2 (3.4–11.2) ^‡^  10.9 (6.2–19.2) ^‡^ | 1  1.4 (0.7–2.6)  1.2 (0.6–2.4)  1.3 (0.7–2.5) | 1  1.3 (0.7–2.5)  1.9 (0.9–3.6)  2.7 (1.5–4.8) ^‡^ |
| Basic health insurance  Private/semi-private | 3.0 (1.0–8.5)  3.0 (1.0–7.5) | 0.43 | 1  1.1 (0.8–1.5) | 1  0.9 (0.6–1.2) | 1  1.0 (0.7–1.3) | 1  0.8 (0.5–1.3) | 1  0.9 (0.6–1.4) |
| Oncological disease | 8.0 (2.0–26) | < 0.01 | 0.3 (0.1–0.6) ^‡^ | 2.9 (1.5–5.8) ^‡^ | 1.1 (0.5–2.3) | 1.9 (0.8–4.8) | 5.4 (2.7–10.7) ^‡^ |
| Cardiovascular disease | 3.0 (1.0–7.5) | 0.81 | 1.1 (0.8–1.6) | 0.8 (0.5–1.3) | 1.5 (1.0–2.2) ^†^ | 0.8 (0.4–1.5) | 0.7 (0.4–1.3) |
| Thromboembolism | 2.0 (1.0–7.5) | 0.33 | 4.6 (1.6–13.2) ^‡^ | 0.3 (0.1–1.1) | 0.5 (0.2–1.3) | 1.7 (0.6–4.5) | 0.7 (0.2–2.2) |
| Stroke | 2.0 (1.0–4.0) | 0.05 | 1.5 (0.8–2.7) | 1.1 (0.5–2.1) | 0.4 (0.2–0.9) ^‡^ | 0.6 (0.2–1.9) | 0.5 (0.1–1.3) |
| Pulmonary disease | 3.0 (1.0–8.5) | 0.45 | 1.1 (0.7–1.7) | 0.9 (0.5–1.6) | 1.1 (0.7–1.8) | 1.6 (0.8–3.2) | 1.3 (0.7–2.4) |
| Infectious disease | 3.0 (1.0–8.0) | 0.40 | 0.9 (0.7–1.3) | 1.1 (0.7–1.6) | 0.9 (0.6–1.4) | 1.1 (0.6–1.8) | 0.7 (0.4–1.2) |
| Abdominal disease | 4.0 (1.0–8.5) | 0.27 | 0.9 (0.6–1.4) | 0.8 (0.4–1.5) | 1.1 (0.7–1.9) | 1.4 (0.6–2.8) | 0.9 (0.5–1.8) |
| Metabolic disease | 2.0 (2.0–6.5) | 0.63 | 0.9 (0.6–1.7) | 1.4 (0.7–2.6) | 0.8 (0.4–1.4) | 0.9 (0.4–2.4) | 0.8 (0.3–1.9) |
| * Kruskal–Wallis test; † p value < 0.05 in unadjusted analysis, but not statistically significant in age- and sex-adjusted analysis; ‡ *p*-value < 0.05 in unadjusted and in age and sex adjusted analysis  PCP = primary care physician; HUTIL = Healthcare Utilisation; LOS = Length of stay; ED = Emergency department | | | | | | | |

| **eTable 4:** 30-day formal support utilization after index hospitalization and their association with potential risk factors. | | | | | | | | | | |
| --- | --- | --- | --- | --- | --- | --- | --- | --- | --- | --- |
|  | **Support (binary)** | | **Support cleaning** | | **Buy grocery** | | **Support for eating** | | **Informal support** | |
|  | n (%) | P value | n (%) | P value | n (%) | P value | n (%) | P value | n (%) | P value |
| Female  Male | 150 (37%)  118 (22%) | <0.01 | 146 (36%)  114 (22%) | <0.01 | 47 (11%)  47 (9%) | 0.23 | 48 (12%)  35 (7%) | <0.01 | 239 (59%)  63 (73%) | <0.01 |
| Age <60 years  Age 61-70 years  Age 71-80 years  Age >80 years | 20 (8%)  54 (22%)  73 (33%)  121 (53%) | <0.01 | 20 (8%)  51 (21%)  69 (31%)  120 (53%) | <0.01 | 8 (3%)  17 (7%)  19 (8%)  50 (22%) | <0.01 | 5 (2%)  13 (5%)  18 (8%)  47 (21%) | <0.01 | 171 (73%)  175 (71%)  155 (70%)  119 (53%) | <0.01 |
| Living alone  Living with someone | 129 (42%)  136 (22%) | <0.01 | 124 (40%)  133 (21%) | <0.01 | 35 (6%)  58 (19%) | <0.01 | 33 (5%)  48 (16%) | <0.01 | 0 (0%)  620 (100%) | <0.01 |
| HOSPITAL score 0-1  HOSPITAL score 2-3  HOSPITAL score 4-5  HOSPITAL score >5 | 40 (23%)  76 (22%)  85 (32%)  67 (46%) | <0.01 | 40 (23%)  74 (21%)  83 (31%)  63 (43%) | <0.01 | 13 (7%)  26 (7%)  34 (13%)  21 (14%) | 0.03 | 6 (3%)  20 (6%)  34 (13%)  23 (16%) | <0.01 | 122 (71%)  234 (68%)  169 (63%)  95 (65%) | 0.36* |
| LOS 0-3 days  LOS 4-5 days  LOS 6-8 days  LOS >8 days | 33 (17%)  53 (23%)  80 (30%)  87 (42%) | <0.01 | 33 (17%)  50 (22%)  77 (29%)  85 (41%) | <0.01 | 5 (3%)  18 (8%)  25 (9%)  39 (19%) | <0.01 | 5 (3%)  16 (7%)  22 (8%)  31 (15%) | <0.01 | 135 (72%)  157 (70%)  176 (66%)  127 (62%) | 0.14 |
| Comorbidities nb=0  Comorbidities nb=1  Comorbidities nb=2  Comorbidities nb=3 | 38 (16%)  69 (27%)  65 (35%)  94 (40%) | <0.01 | 37 (16%)  68 (26%)  63 (33%)  90 (38%) | <0.01 | 8 (3%)  23 (9%)  27 (14%)  35 (15%) | <0.01 | 7 (3%)  20 (8%)  20 (11%)  35 (15%) | <0.01 | 187 (80%)  168 (66%)  109 (58%)  144 (62%) | <0.01 |
| Standard Insurance  Standard +  Private / semi private | 97 (22%)  102 (38%)  69 (30%) | <0.01 | 92 (21%)  100 (37%)  68 (30%) | <0.01 | 40 (9%)  28 (10%)  26 (11%) | 0.66 | 26 (6%)  34 (13%)  23 (10%) | <0.01 | 276 (64%)  174 (65%)  169 (74%) | 0.02 |
| Nb: number; PCP = primary care provider.  * Fisher exact test | | | | | | | | | | |

| **eTable 5:** 30-day age- and sex- adjusted Odd ratios for formal support utilization after index hospitalization and their association with potential risk factors. | | | | |
| --- | --- | --- | --- | --- |
|  | **Support (binary)**  OR (95%CI) | **Support cleaning**  OR (95%CI) | **Buy grocery**  OR (95%CI) | **Support for eating**  OR (95%CI) |
| Male  Female | -  1.8 (1.3-2.4)* | -  1.8 (1.3-2.4)* | -  1.1 (0.7-1.7) | -  1.5 (0.9-2.4) |
| Age <60 years  Age 61-70 years  Age 71-80 years  Age >80 years | -  3.0 (1.8-5.3)*  5.2 (7.0-8.9)*  11.8 (7.0-20.1)* | -  2.8 (1.6-4.9)*  4.7 (2.8-8.2)*  11.6 (6.8-19.7)* | -  2.1 (0.9-5.0)  2.6 (1.1-6.2)*  8.0 (3.7-17.3)* | -  2.6 (0.9-7.3)  3.9 (1.4-10.8)*  11.4 (4.4-29.4)* |
| Living with someone  Living alone | -  0.5 (0.4-0.7)* | -  0.5 (0.4-0.7)* | -  0.3 (0.2-0.5)* | -  0.4 (0.3-0.7)* |
| LOS 0-3 days  LOS 4-5 days  LOS 6-8 days  LOS >8 days | -  1.3 (0.8-2.2)  1.6 (0.9-2.6)  2.6 (1.5-4.2)* | -  1.2 (0.7-2.1)  1.5 (0.9-2.5)  2.4 (1.5-4.1)* | -  3.0 (1.1-8.4)  3.0 (1.1-8.1)  6.2 (2.3-16.3)* | -  2.7 (0.9-7.7)  2.6 (0.9-7.1)  4.7 (1.7-12.5)* |
| HOSPITAL score 0-1  HOSPITAL score 2-3  HOSPITAL score 4-5  HOSPITAL score >5 | -  0.8 (0.5-1.3)  1.2 (0.7-1.9)  2.3 (1.4-4.0)* | -  0.8 (0.5-1.3)  1.1 (0.7-1.9)  2.1 (1.2-3.5)* | -  0.8 (0.4-1.9)  1.6 (0.7-2.8)  1.6 (0.8-3.5) | -  1.6 (0.6-4.3)  3.5 (1.4-8.7)*  4.5 (1.7-11.8)* |
| Comorbidities nb=0  Comorbidities nb=1  Comorbidities nb=2  Comorbidities nb=3 | -  1.6 (0.9-2.6)  2.0 (1.2-3.4)*  2.1 (1.3-3.4)* | -  1.6 (0.9-2.6)  2.0 (1.2-3.3)*  2.0 (1.2-3.2)* | -  2.2 (0.9-5.1)  3.3 (1.4-7.5)*  2.7 (1.2-6.2)* | -  2.2 (0.9-5.5)  2.7 (1.1-6.7)*  3.3 (1.4-7.9)* |
| Standard Insurance  Standard +  Private / semi private | -  1.8 (1.3-2.6)*  1.1 (0.7-1.6) | -  1.9 (1.3-2.7)*  1.1 (0.8-1.7) | -  0.9 (0.5-1.6)  0.9 (0.5-1.6) | -  1.9 (1.1-3.3)*  1.3 (0.7-2.4) |
| ER = emergency room; GP: general practitioner; Nb: number; OR: odds ratio; PCP = primary care provider (GP or specialist);  *p value <0.05 | | | | |

| **Table 6:** 30-day formal support utilization and its association with main diagnosis category of the index hospitalization. | | | | | |
| --- | --- | --- | --- | --- | --- |
|  | | **Support (binary)**  OR (95%CI) | **Support cleaning**  OR (95%CI) | **Buy grocery**  OR (95%CI) | **Support for eating**  OR (95%CI) |
| Cardiovascular | **OR**  **aOR** | 1.2 (0.8-1.8)  0.9 (0.6-1.3) | 1.2 (0.8-1.7)  0.9 (0.6-1.3) | 1.3 (0.8-2.3)  1.0 (0.6-1.7) | 1.2 (0.7-2.2)  0.9 (0.5-1.6) |
| Thrombosis and pulmonary embolism | **OR**  **aOR** | 0.6 (0.3-1.5)  0.7 (0.3-1.8) | 0.7 (0.3-1.5)  0.8 (0.3-1.9) | 0.3 (0.1-1.9)  0.3 (0.1-2.5) | 0.6 (0.1-2.7)  0.8 (0.2-3.6) |
| Stroke | **OR**  **aOR** | 0.8 (0.4-1.6)  0.8 (0.4-1.5) | 0.8 (0.4-1.5)  0.7 (0.4-1.4) | 0.2 (0.1-1.1)  0.2 (0.1-1.1) | 0.4 (0.1-1.6)  0.3 (0.1-1.5) |
| Pulmonary disease | **OR**  **aOR** | 1.4 (0.9-2.3)  1.5 (0.9-2.4) | 1.4 (0.9-2.2)  1.4 (0.9-2.4) | 1.2 (0.6-2.4)  1.1 (0.5-2.2) | 1.2 (0.6-2.5)  1.2 (0.5-2.5) |
| Infectious disease | **OR**  **aOR** | 1.1 (0.8-1.6)  1.3 (0.9-2.0) | 1.1 (0.8-1.6)  1.4 (0.9-2.0) | 0.8 (0.5-1.4)  0.9 (0.5-1.6) | 0.8 (0.5-1.5)  0.9 (0.5-1.7) |
| Abdominal disease | **OR**  **aOR** | 1.4 (0.9-2.3)  1.5 (0.9-2.7) | 1.5 (0.9-2.4)  1.6 (0.9-2.8) | 1.2 (0.6-2.5)  1.3 (0.6-2.8) | 1.8 (0.9-3.7)  2.0 (0.9-4.2) |
| Metabolic disease | **OR**  **aOR** | 0.6 (0.3-1.1)  0.6 (0.3-1.3) | 0.5 (0.3-1.1)  0.6 (0.3-1.2) | 0.8 (0.3-2.2)  1.0 (0.4-2.5) | 0.5 (0.2-1.8)  0.6 (0.2-2.1) |
| Oncologic disease | **OR**  **aOR** | 0.7 (0.3-1.6)  1.1 (0.4-2.6) | 0.6 (0.3-1.5)  0.9 (0.4-2.3) | 1.1 (0.4-3.4)  1.7 (0.6-5.4) | 1.7 (0.7-4.6)  3.1 (1.1-8.8)* |
| * p value <0.05; aOR: adjusted odds ratio ER = emergency room; PCP: primary care physician; OR: odds ratio | | | | | |

| **eTable 7:** Modified HUTIL index* and mortality beyond 30-day after discharge (sensitivity analyses). | | | | |
| --- | --- | --- | --- | --- |
| **Modified HUTIL index** | **HR** | **P value** | **Adjusted HR** | **P value** |
| **0-2.9 (ref.)**  **3-5.9**  **6-8.9**  **≥9** | 1  1.3 (0.7-2.4)  4.1 (1.9-8.9)  5.0 (3.0-8.2) | 0.48  <0.001  <0.001 | 1  1.4 (0.7-2.6)  4.9 (2.2-10.7)  5.1 (3.2-8.5) | 0.36  <0.001  <0.001 |
| * including only one visit for patients with known nurse visits at their home. | | | | |
